# Impact of urban spatial structure elements on carbon emissions efficiency in growing megacities: the case of Chengdu

**DOI:** 10.1038/s41598-023-36575-6

**Published:** 2023-06-19

**Authors:** Tian Feng, Bo Zhou

**Affiliations:** College of Architecture and Environment, Sichuan University, Chengdu, 610065 China

**Keywords:** Climate-change mitigation, Energy and society, Environmental impact, Sustainability

## Abstract

Quantitative research on the impact weight and impact of regional heterogeneity of urban spatial structure elements on carbon emissions efficiency can provide a scientific basis and practical guidance for low-carbon and sustainable urban development. This study uses the megacity of Chengdu as an example to measure and analyze the spatial carbon emission efficiency and multidimensional spatial structure elements by building a high-resolution grid and identifying the main spatial structure elements that affect urban carbon emissions and their impact weights via the Ordinary Least Squares regression (OLS) and Geographically Weighted Regression (GWR). The spatial heterogeneity of the impact of each element is also explored. The results show that the overall carbon emission efficiency of Chengdu is high in the center and low on the sides, which is related to urban density, functional mix, land use, and traffic structure. However, the influence of each spatial structure element is different in the developed central areas, developing areas of the plain, mountainous developing areas, underdeveloped areas of the plain, and mountainous underdeveloped areas. Thus, it is appropriate to form differentiated urban planning strategies based on the characteristics of the development of each zone. The findings provide inspiration and a scientific basis for formulating policies and practice to the future low-carbon development of Chengdu, while provide a reference for other growing megacities.

## Introduction

Since opening up in 1978, China's accelerated urban development and rapid growth of population have caused a surge in carbon emissions. As the main drivers of economic growth, cities consume large amounts of energy, their consumption exceeding 70% of the total; this figure is predicted to rise in developing nations in the future^1^. Therefore, the transition toward low-carbon urban planning is necessary to facilitate a sustainable future. This requires a deeper understanding of the influence of multidimensional spatial elements on urban carbon emissions.

Existing studies on low-carbon urban planning comprise multilevel elements of urban spatial structure. In terms of urban structure and land use, several scholars have suggested that compact and high-density city has a positive effect on reducing carbon emissions. Evidence from cities in both developed and developing countries suggests that compact cities have a positive effect on reducing transportation carbon emissions^2,3^. Residential carbon emissions are also been shown to be closely related to urban structure and land use^4^. The urban form is a significant factor related to overall heating energy demand and individual dwelling energy consumption for space heating^5^. Sector-wide carbon emissions are affected by urban structure and land use as well. A study in Guangdong-Hong Kong-Macao Greater Bay Area shows there is a link between the spatial structure, land use, and carbon emission efficiency of cities^6^.

Urban traffic structure is another essential influence on carbon emissions. Several studies have pointed out that road conditions, road network density, and transportation facilities are important factors that affect carbon emissions from travel^7–9^. Province-level research supports that urban road density and per capita highway mileage are strongly connected to transportation carbon emissions^10^. Neighborhood-level indicates transit accessibility, bus stop density, metro station density, and road network density have an impact on carbon emissions^7–9^. There is variability in the findings of some indicators between the province-level and community-level studies, which suggests that different results may exist in studies at different scales.

As an important element in urban spatial structure, urban density has an obvious impact on carbon emissions. Population density is strongly associated with carbon emissions^11^, but its impact may be influenced by different levels of the urbanization process^12^. In addition, job density, land use density, and so on are also thought to have an impact on carbon emissions^9^.

Existing studies suggest that there is a relationship between different spatial structure elements and carbon emissions, but there are two aspects that remain less covered. First, most studies have focused on carbon emissions from a single sector or a single category of structural elements, thus, lacking a composite analysis of multilevel spatial structure, which makes it difficult to compare the influence of each category. Second, the studies were mostly conducted on larger urban clusters or smaller communities; thus, it is difficult to explore the different influence of elements in different development levels of zones within a single city. Therefore, this study takes all sector carbon emissions and multidimensional spatial structure elements into account, trying to identify the most influential spatial structure elements on carbon emissions, and explore in depth the ways in which each element is influenced. The study divides Chengdu into grids to fully discuss the spatial heterogeneity in the effect of each element by zone.

Chengdu, a fast-growing megacity, is a nationally central city in western China. The Chinese megacities are still continuously growing in size of population and infrastructure, causing surges in carbon emissions. At this stage of carbon emission surge, formulating reasonable measures to improve carbon emission efficiency is extremely effective in reducing carbon emissions. From the perspective of sustainable development, to maintain growth while also reducing their environmental footprint, megacities like Chengdu must pay attention to the planning process. Taking Chengdu as an example, this study explores the mechanism of how multidimensional spatial elements influence carbon emission efficiency in fast-growing megacities. The study broadens the scope of carbon emission research in the urban setting, enabling an effective layout of spatial elements for a low-carbon urban environment by providing supporting data and theory. It can also provide a reference for other fast-growing megacities while promoting the low-carbon development of Chengdu in the future.

## Methods

The study is divided into four sections: analysis of carbon emissions efficiency characteristics, measurement and analysis of urban spatial structure elements, construction of a model of the relationship between multilevel spatial structure elements and carbon emission efficiency, and suggestions for promoting urban green development. This study aims to explain the intrinsic relative relationship between the multilevel urban spatial structure elements and carbon emissions in different zones of Chengdu, based on objective and quantitative expressions of carbon emission efficiency characteristics and urban spatial elements. The study also has the objective of identifying the characteristics presented by the influence of different elements in spatial heterogeneity and then proposing spatial planning intervention measures and policies. The main methods and data sources for each part are described in the relative sections. All data period in this study is 2019.

### Study area

**Figure 1:**
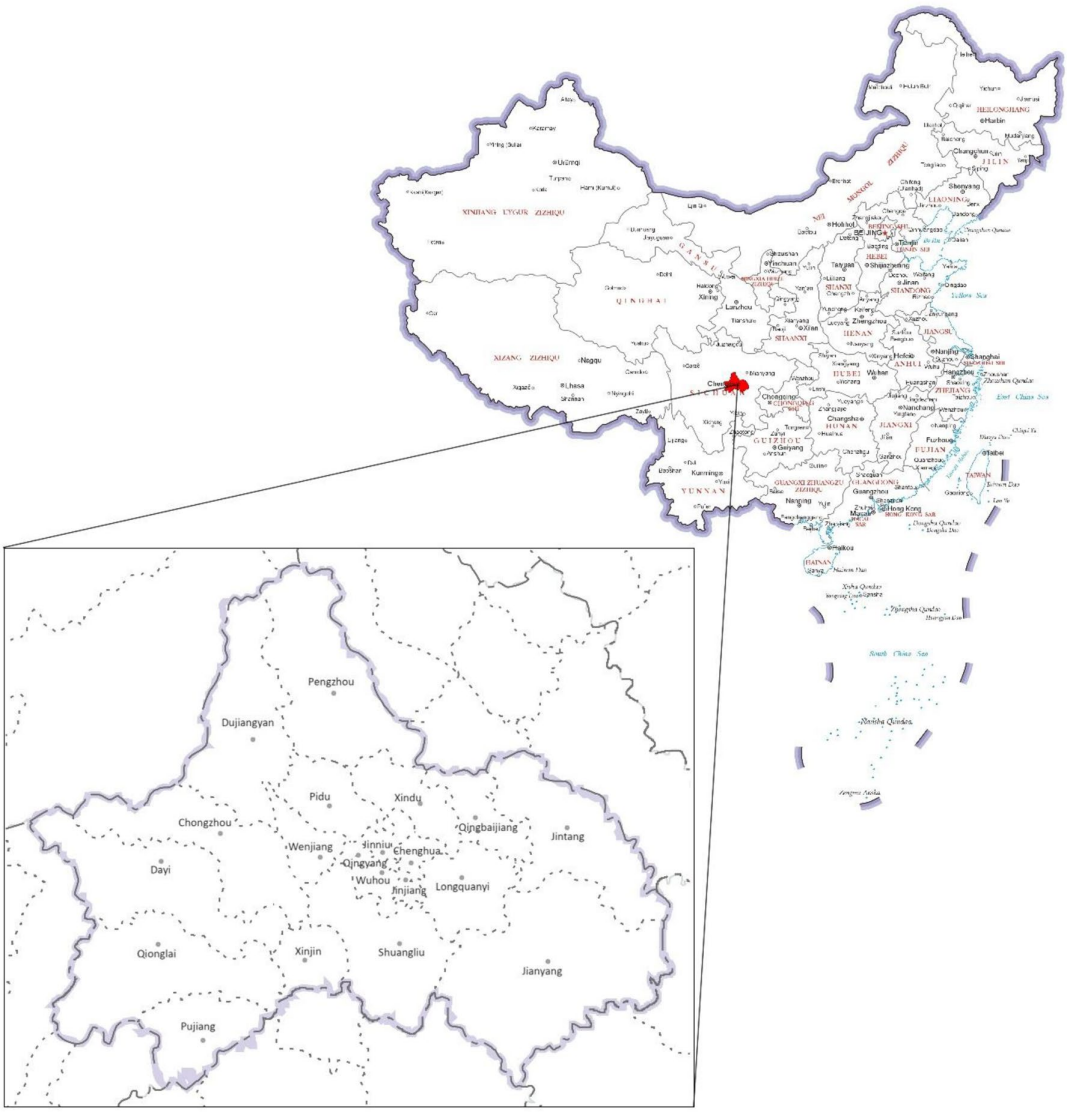
The location and geographic scope of Chengdu. (Note: the map on the right side is from Ministry of Natural Resources of the People’s Republic of China, No.GS(2019)1671, http://bzdt.ch.mnr.gov.cn/browse.html?picId=%224o28b0625501ad13015501ad2bfc0266%22; the map on left side was drawn by author with ArcGIS Pro, Version 3.0.2, ESRI, referring to information on Sichuan Bureau of Surveying, Mapping and Geoinformation, http://scsm.mnr.gov.cn/nbzdt.htm.)

Chengdu, a fast-growing city in China, is selected as the study area (Fig. 1). As the provincial capital of Sichuan Province, Chengdu is one of the first national historical and cultural cities in China, and an important central city in southwest China, which was selected as a low-carbon pilot city in China in 2017. Chengdu has 12 districts of Jinjiang, Qingyang, Jinniu, Wuhou, Chenghua, Longquanyi, Qingbaijiang, Xindu, Wenjiang, Shuangliu, Pidu and Xinjin, 5 county-level cities of Jianyang, Dujiangyan, Pengzhou, Qionglai and Chongzhou, and 3 counties of Jintang, Dayi and Pujiang. As of 2020, Chengdu has a total area of 14,335 square kilometers, a resident population of 20,937,800, and a total GDP of more than 1.7 trillion yuan. In recent years, Chengdu has enjoyed good economic development and has been ranked among the top ten cities in China in terms of total GDP since 2011.

Chengdu has a long history of development, with heterogeneity in construction and variability in urban structure elements in different regions, which provides an ideal sample for studying the impact of urban spatial structure elements on carbon emissions efficiency. As a low-carbon pilot city in China, it has comparatively sufficient conditions for the practical promotion of the study results.

### Analysis of carbon emission efficiency characteristics

**Figure 2:**
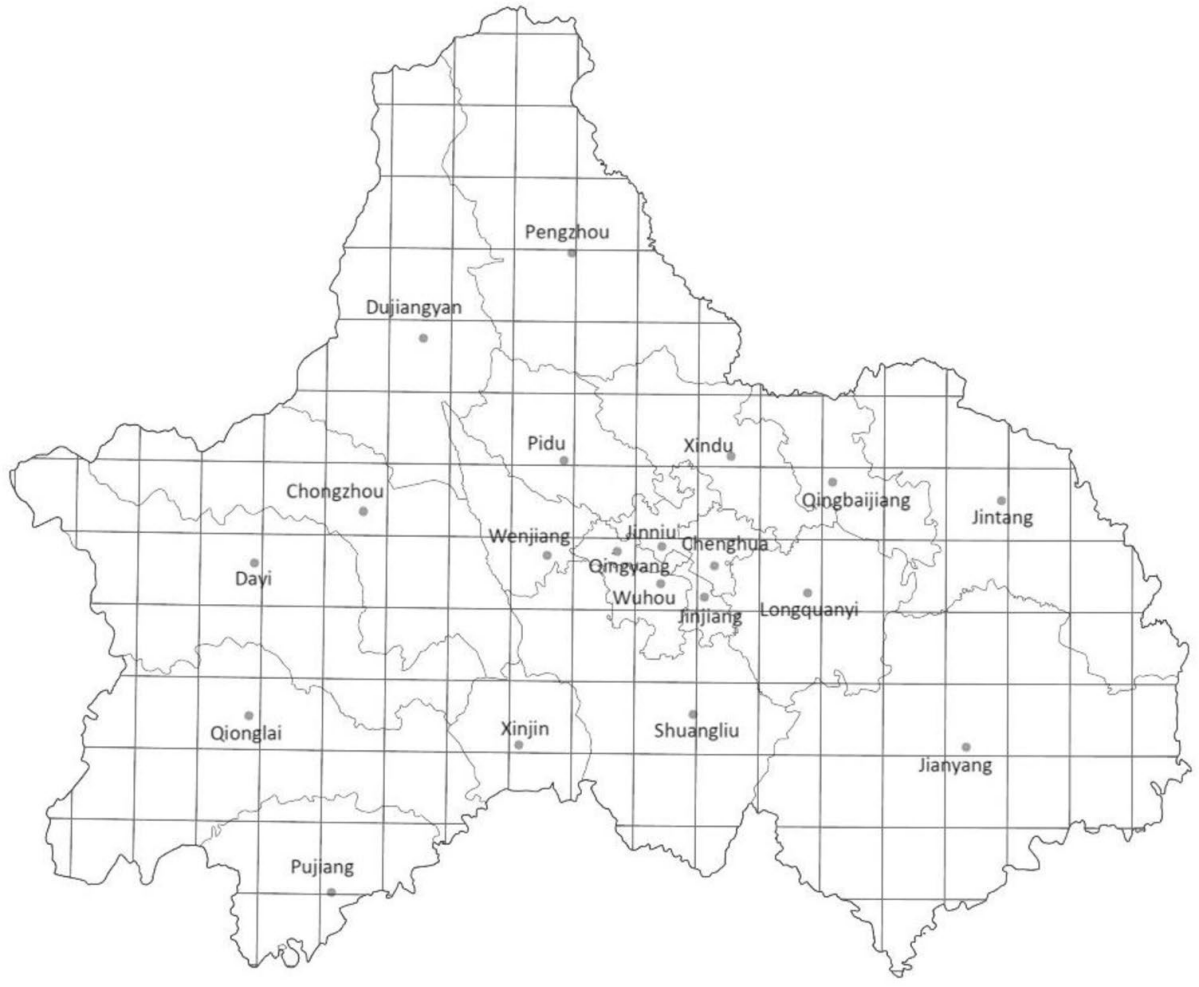
The intra-city carbon emission efficiency grid of the city of Chengdu, which is a 0.1° × 0.1° grid divided by latitude and longitude, with an actual area of approximately 10 km × 10 km, 176 samples in total. (Note: ArcGIS Pro, Version 3.0.2, ESRI was used to create this figure).

Most existing studies estimate carbon emissions through energy consumption data at the provincial, municipal, and county levels^13,14^. These estimates lack precision in characterizing carbon emissions within cities. There are significant differences in the internal carbon emission levels of each unit area within a city owing to their development level variability^15^. Therefore, to fully reflect the intra-city geographic carbon emission differences, this study developed a high-resolution intra-city carbon emission efficiency grid (0.1° × 0.1° grid divided by latitude and longitude, with an actual area of approximately 10 km × 10 km, 176 samples in total, Fig. 2) for carbon emission efficiency characteristics analysis. The total carbon emission data were obtained using the base point source data from the Global Energy Infrastructure Emissions Database^16^, and the GDP data were obtained from the Chinese GDP spatial distribution dataset(1 km × 1 km) from the Resources and Environmental Science Data Center of the Chinese Academy of Sciences(RESDC)^17^.

Carbon emission efficiency generally refers to achieving higher economic growth with lower carbon dioxide emissions^18^. In this study, carbon emission efficiency was measured and analyzed by carbon emissions per unit of GDP (kgCO_2_/10,000 yuan) (indicated by CBN in subsequent tables and pictures). The higher the carbon emissions per unit of GDP, the lower the carbon emission efficiency.

### Measurement and analysis of spatial structure elements

Urban spatial structure refers to the layout of a city's components^19^ and is affected by the interactions of between these components.

In existing studies, several studies have pointed out that urban density^12,20–22^, functional mix^23^, land use type^24,25^, and traffic structure^10,26^ have an impact on the carbon emissions efficiency of cities. Since the scale of this study is within a single city, which is relatively smaller, the study combines 5Ds built environment elements raised by Ewing^27^ to refine the elements for the above four categories, resulting in the 14 elements from four categories in Table 1. This spatial data (roads, buildings, points-of-interest, stations, etc.) was run through AMAP and Bigemap and vectorized through ArcGIS.

**Table 1:** Spatial structure elements.

Category	Elements	Calculation instructions	Units	Original data	Data source	Format
Urban density	Population density (pop)	population per unit grid	pcs	Population	RESDC^33^	Grid (1 km × 1 km with location)
	Building density (bda)	Total area occupied by buildings per unit grid	m^2^	Building area	Bigemap	Shapefile (polygon with location)
	POI density (poi)	Total number of POI per unit grid (pcs)	pcs	POI	AMAP	Shapefile (point with location)
Functional mix	Land use mix (pim)	Measurement of land use mix based on POI categories using the Shannon Diversity Index	–	POI	AMAP	Shapefile (point with location)
	Land nature mix (phh)	Measuring land use assemblages based on land properties using the Shannon Diversity Index (SHDI)	–	Land properties	RESDC^34^	Grid (1 km × 1 km with location)
Land use type	Construction site area (cst)	Construction site area per unit grid	m^2^	Land properties	RESDC^34^	Grid (1 km × 1 km with location)
	Green area (gre)	Green space area per unit grid	m^2^	Land properties	RESDC^34^	Grid (1 km × 1 km with location)
	Water area (wtr)	Water area per unit grid	m^2^	Land properties	RESDC^34^	Grid (1 km × 1 km with location)
	Land patch scale (lsc)	The average size of each different land parcel per unit grid	m^2^	Land properties	RESDC^34^	Grid (1 km × 1 km with location)
Traffic structure	City roads length (crd)	Total length of urban trunk roads, urban secondary roads and urban feeder roads per unit grid	m	City road	Bigemap	Shapefile (line with location)
	Fastway length (fsw)	Total length of urban expressways and elevated roads per unit grid	m	Fastway	Bigemap	Shapefile (line with location)
	Station density (sta)	Number of bus stops and subway stops within the unit grid	pcs	Bus stop and subway station	AMAP	Shapefile (point with location)
	Distance to the nearest transit station (dis)	Distance from the grid center point to the nearest traffic station	m	Transit station	AMAP	Shapefile (point with location)
	Railway length (rlw)	Total railroad length per unit grid	m	Railway	Bigemap	Shapefile (line with location)

### Modeling the relationship between multilevel spatial structure elements and carbon emission efficiency

To model the relationship between various spatial structure elements and carbon emission, Ordinary Least Squares regression (OLS) and Geographically Weighted Regression (GWR) are performed to find a better model.

OLS regression is mainly used for parameter estimation in linear regression, and its goal is to find the best functional match for the data by minimizing the squared error and can be expressed by Eq. (1):

1 $$y={\beta }_{0}+{\beta }_{1}{x}_{1}+{\beta }_{2}{x}_{2}+{\beta }_{3}{x}_{3}+\dots +{\beta }_{n}{x}_{n}+\varepsilon$$

where $y$ is the dependent variable, $x$ is the independent variable, ${\beta }_{0}$ is the const, ${\beta }_{n}$ is the coefficient, $\varepsilon$ is the error term.

GWR is a spatial analysis technique widely used in geography and related disciplines involving spatial pattern analysis. It explores the spatial variation of a study object at a certain scale and the associated drivers by establishing a local regression equation at each point in the spatial range, and can be used to make predictions of future results. It has the advantage of higher accuracy because it takes into account the local effects of spatial objects. GWR can be expressed as shown in Eq. (2):

2 $${y}_{i}={\beta }_{0}\left({u}_{i},{v}_{i}\right)+{\sum }_{m=1}^{p}{\beta }_{m}({u}_{i},{v}_{i}){x}_{im}+{\varepsilon }_{i}$$

where ${y}_{i}$ is the dependent variable of point i, $\left({u}_{i},{v}_{i}\right)$ is the latitude and longitude coordinates of point i, ${\beta }_{m}({u}_{i},{v}_{i})$ is m-th coefficient of the point i, ${x}_{im}$ is the m-th independent variable of the point i, ${\varepsilon }_{i}$ is the error term of point i.

The specific steps are as follows. First, correlation analysis and OLS regression constructs were performed on the spatial structure elements listed in the previous section. The elements significantly influencing carbon emission efficiency were analyzed and screened. Subsequently, the OLS regression model was adjusted to analyze the relative influence, extent, and direction of each indicator on carbon emission efficiency through coefficients. Finally, the spatial heterogeneity of the influence of each element was clarified by further geographically weighted regression analysis, determining the factors and principle of change in the extent of influence.

## Results and discussion

### Carbon emission efficiency characterization

**Figure 3:**
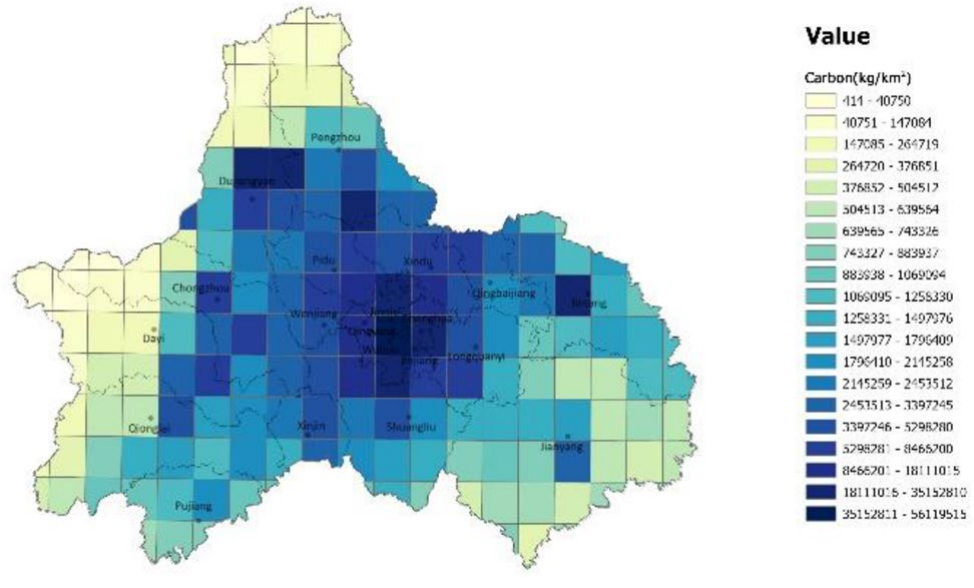
A description of the spatial distribution of carbon emissions in Chengdu (unit: kg/km^2^). The color range spanning from blue to yellow, represents the carbon emissions from high to low. (Note: ArcGIS Pro, Version 3.0.2, ESRI was used to create this figure).

**Figure 4:**
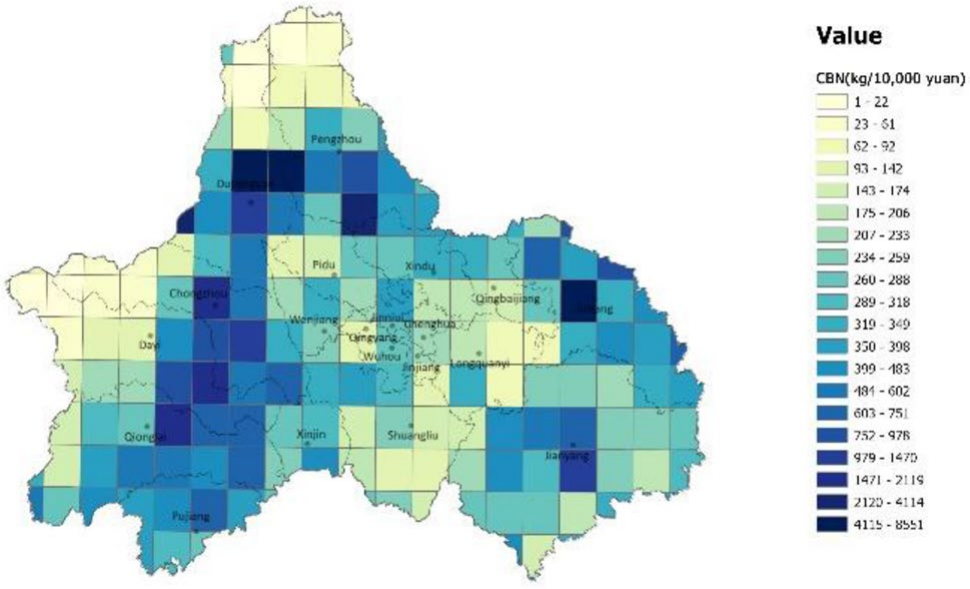
A description of the spatial distribution of carbon emissions efficiency in Chengdu (unit: kg/10,000 yuan). The color range spanning from blue to yellow, represents the carbon emissions from high to low. (Note: ArcGIS Pro, Version 3.0.2, ESRI was used to create this figure).

A high-resolution carbon emission efficiency grid of Chengdu city was constructed using a data overlay, and the spatial distribution of carbon emission efficiency in Chengdu city was obtained by grading according to natural interruption points (Figs. 3, 4). According to the constructed grid, the area with the highest carbon emission efficiency (lowest CBN value) is the western mountainous area (the northern part of Dujiangyan and Pengzhou City, the western part of Dayi County, Chongzhou City, and Qionglai City), followed by central city. The southeastern (Jinyang City and Jintang County) and western plain regions (Dujiangyan City and southern Pengzhou City, Dayi County, Chongzhou City, and eastern Qionglai City) had lower overall carbon emission efficiency.

**Figure 5:**
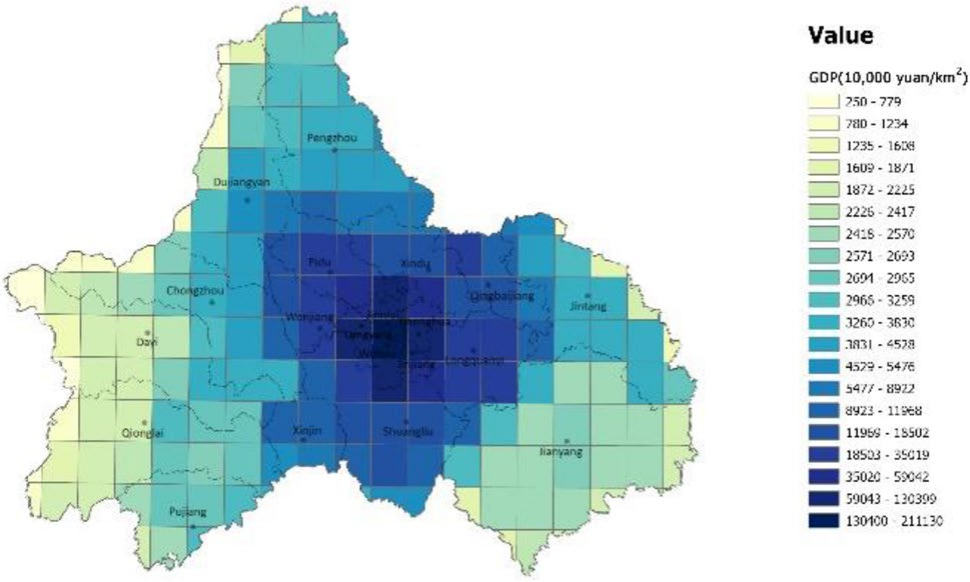
A description of the spatial distribution of GDP in Chengdu (unit: 10,000 yuan/ km^2^). The color range spanning from blue to yellow, represents the carbon emissions from high to low. (Note: ArcGIS Pro, Version 3.0.2, ESRI was used to create this figure).

The aggregate carbon emission efficiency was analyzed using the spatial distribution map of GDP (Fig. 5) and the topographical conditions of Chengdu. In the central urban area, the structure density is high, particularly in the high-tech zone. The overall demand for energy and transportation in such areas is relatively small, and the high per unit GDP translates to higher carbon emission efficiency. It is worth noting that the eastern part of the central city has a strip-like area with very high carbon emission efficiency, the Longquan Mountain Range, which is characterized by woodlands that are effective carbon sinks. Overall, urban high-density areas and ecological areas are more carbon efficient than other regions, which is consistent with the study of Li^6^.

### Measurement and analysis of spatial structure elements

**Figure 6:**
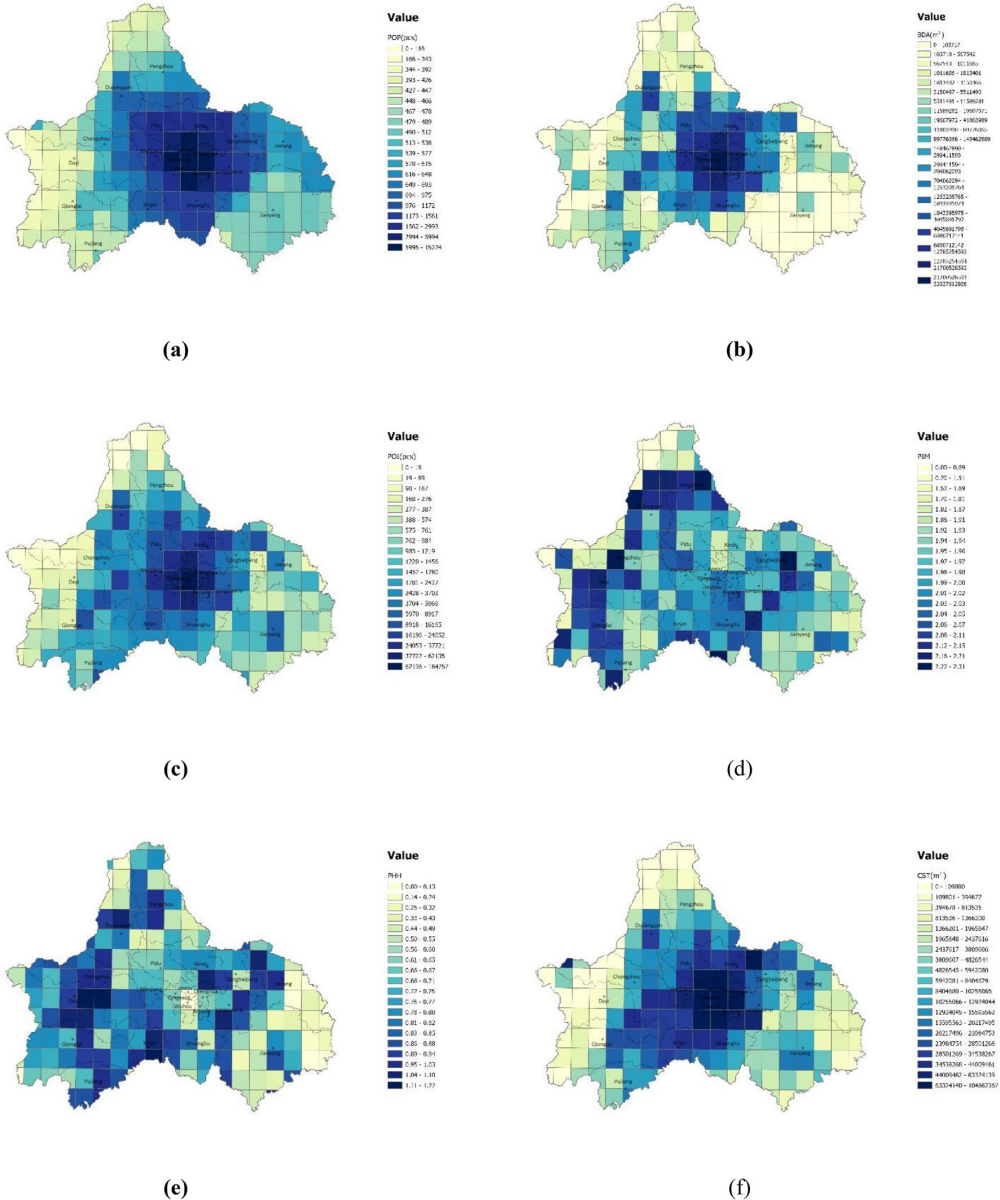

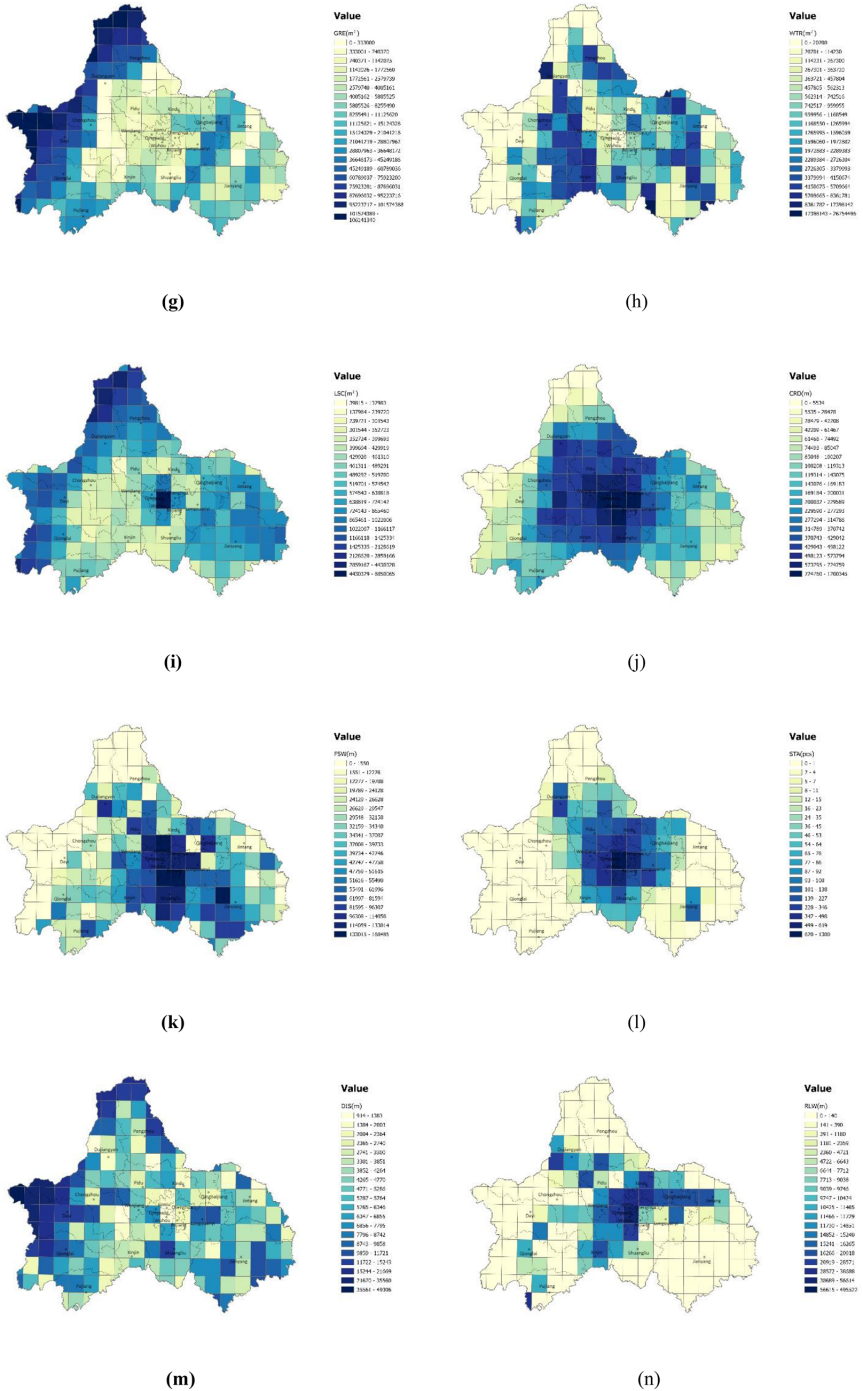
Spatial distribution of spatial structure elements of Chengdu: (**a**) Population density; (**b**) Building density; (**c**) POI density; (**d**) Land use mix; (**e**) Land nature mix; (**f**) Construction site area; (**g**) Green area; (**h**) Water area; (**i**) Land patch scale; (**j**) City roads length; (**k**) Fastway length; (**l**) Station density; (**m**) Distance to the nearest transit station; (**n**) Railway length. The color range spanning from blue to yellow, represents values from high to low. (Note: ArcGIS Pro, Version 3.0.2, ESRI was used to create this figure).

After obtaining and adjusting the data related to urban density, functional mix, traffic structure, and land use type in Chengdu, the study adopted the spatial structure element projection based on the high-resolution grid of the city to obtain the specific grid data of each element. the results are shown in Fig. 6.

The highest values of population density, building density, POI density, construction site area, city road length, station density, and railway length appear in the center of the city, and then decrease from the central city to the surrounding area in different trends: the low population density appears in the southwest; low building density is mainly concentrated in the southeast; low POI density appears in the west and north; low construction site area and city road length are concentrated in the northwest and southeast; stations are mainly concentrated in the central city, with less coverage around; railways are more scattered in the city. Both green area and land patch scale are lower in the central city, with the highest values of the former in the west and north, and the latter in the northwest. Land use mix and land nature mix are found high in the Min Mountain area in the west and Longquan Mountain area in the east, while the lowest land use mix value in the north, and the lowest land nature mixture value in the southeast. The values of water area and distance to the nearest transit are distributed randomly.

### Identification of key urban spatial structure elements affecting carbon emission efficiency

Before model construction, the study performed a data cleaning and conducted a logarithmic transformation of the elements with larger values to ensure the relative stability of the data. The final analysis sample size is 172. The results of the Pearson correlation analysis of carbon emissions per unit of GDP and each spatial structure element through SPSS R26.0.0.0 are presented in Table 2. The results show that, except for population density and bus stop density, all elements of spatial structure elements show significance at 0.01 (two-sided) level with carbon emissions per unit of GDP, among which green space and land patch scale are negatively correlated (positive correlation with carbon emission efficiency), and the remaining elements show a positive correlation with carbon emissions per unit GDP (negative correlation with carbon emission efficiency).

**Table 2:** Pearson correlation analysis result.

	lg_cbn
lg_pop	0.153*
lg_poi	0.559**
lg_cst	0.421**
lg_gre	− 0.270**
lg_wtr	0.455**
lg_lsc	− 0.222**
lg_bda	0.304**
lg_rlw	0.205**
lg_fsw	0.466**
lg_crd	0.559**
lg_sta	0.153*
lg_dis	− 0.530**
pim	0.479**
phh	0.207**

*p < 0.05, **p < 0.01.

The correlation analysis mainly shows the degree of closeness between two variables, and it is not yet possible to identify the multivariate interactions and interdependencies. Therefore, further OLS regression analysis was required to examine the extent and direction of the influence of each element on carbon emission efficiency. Therefore, we constructed OLS regression models for the analysis (Table 3). The regression results passed the F-test (p < 0.05), and the adjusted R-squared was 0.555, showing a good fit.

**Table 3:** OLS regression analysis result for all elements.

	Coef	Std. err	t	p	VIF
const	4.850	0.884	5.484	0.000**	–
lg_pop	− 0.567	0.167	− 3.393	0.000**	3.870
lg_poi	0.333	0.067	5.006	0.000**	7.646
lg_cst	− 0.053	0.019	− 2.771	0.006**	3.214
lg_gre	− 0.040	0.02	− 1.922	0.056	1.695
lg_wtr	0.004	0.015	0.270	0.787	2.496
lg_lsc	0.016	0.109	0.150	0.881	1.468
lg_bda	− 0.009	0.012	− 0.723	0.471	3.028
lg_rlw	0.004	0.019	0.120	0.842	1.825
lg_fsw	0.053	0.021	2.511	0.013*	2.712
lg_crd	0.174	0.037	4.744	0.000**	4.813
lg_sta	− 0.144	0.053	− 2.686	0.008**	3.577
lg_dis	− 0.449	0.105	− 4.288	0.000**	1.775
pim	− 0.376	0.106	− 3.558	0.001**	5.629
phh	0.248	0.144	1.722	0.087	2.000
R^2^	0.591				
AdjR^2^	0.555				
F	16.221, p = 0.000*				
AICc	150.523				

y:lg_cbn.

*p < 0.05, **p < 0.01.

As seen from Table 4, POI density, expressway length, and urban road length have a significant positive influence on carbon emissions per unit of GDP (negatively correlated with carbon emission efficiency). The relative weight of influence was as follows: POI density > city roads length > fastway length. This indicates that increasing the supply of urban roads would promote emissions, which is inconsistent with the study in traffic carbon emissions^10^.

**Table 4:** OLS regression analysis result for effective elements.

	Coef	Std. Err	t	p	VIF
const	4.951	0.627	7.890	0.000**	–
lg_pop	− 0.651	0.159	− 4.084	0.000**	3.542
lg_poi	0.351	0.060	5.871	0.000**	6.190
lg_cst	− 0.044	0.018	− 2.478	0.014*	2.825
lg_fsw	0.056	0.018	3.111	0.002**	2.011
lg_crd	0.179	0.035	5.054	0.000**	4.497
lg_sta	− 0.132	0.053	− 2.519	0.013*	3.424
lg_dis	− 0.448	0.102	− 4.401	0.000**	1.684
pim	− 0.395	0.102	− 3.885	0.000**	5.254
R^2^	0.577				
AdjR^2^	0.557				
F	27.828, p = 0.000*				
AICc	142.148				

y:lg_cbn.

*p < 0.05, **p < 0.01.

Population density, construction site area, station density, distance to the nearest transit station, and land use mix had a significant negative effect on carbon emissions per unit of GDP (positively correlated with carbon emission efficiency); the relative weights of impact were as follows: population density > distance to the nearest transit station > land use mix > station density > construction site area. The findings are generally consistent with the finding of former studies^7,9,11,12^. However, the effect of the construction site area is different from Liu’s^11^, which may be attributed to the flat topography, concentric type expansion, and strong transportation system of Chengdu, making the increase of various transportation energy consumption insignificant.

However, building density, land nature mix, green area, water area, railway length, and land patch scale did not affect carbon emissions per unit of GDP. Green land, considered significant in some studies^25^, does not show significance in this study, which may be related to the size and degree of development of the city, and presumably because its effect has been captured by the other variables.

### Spatial heterogeneity in the influence of urban spatial structure elements

The eight effective urban spatial structure elements identified above were regressed with OLS again. All elements were determined to be significant after regression (Table 4), and the positive and negative relationships with carbon emissions per unit of GDP were consistent with the results of previous regression analyses. The adjusted R-squared was 0.557.

**Figure 7:**
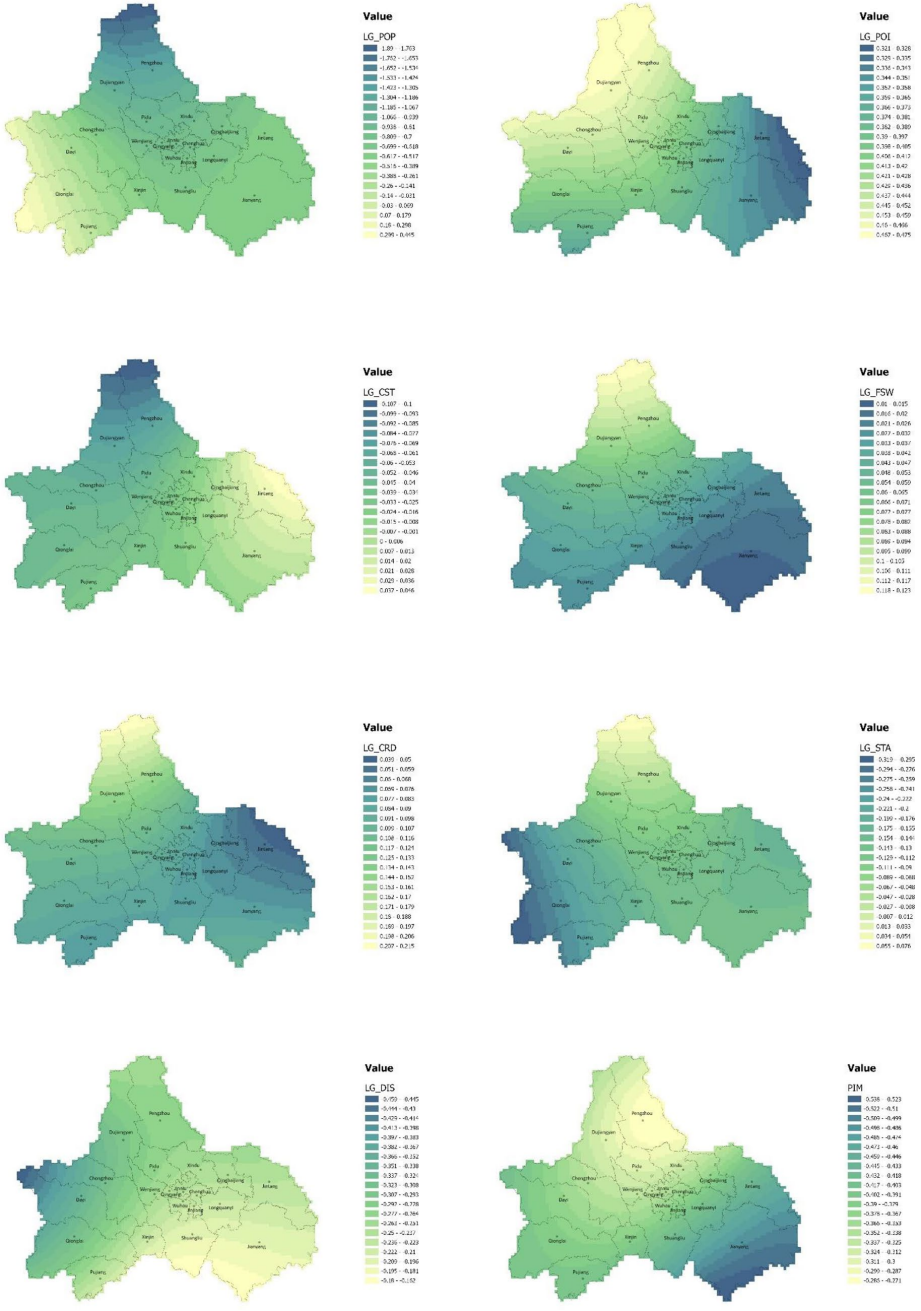
Spatial distribution of influence coefficients of spatial structure elements: (**a**) Population density; (**b**) Building density; (**c**) POI density; (**d**) Land use mix; (**e**) Land nature mix; (**f**) Construction site area; (**g**) Green area; (**h**) Water area; (**i**) Land patch scale; (**j**) City roads length; (**k**) Fastway length; (**l**) Station density; (**m**) Distance to the nearest transit station; (**n**) Railway length. The color range spanning from yellow to green, represents values from high to low. (Note: ArcGIS Pro, Version 3.0.2, ESRI was used to create this figure).

However, the OLS regression cannot depict the spatial nonstationary characteristics of the elements^28^. Existing studies has proved that the GWR model is considered more appropriate to estimate parameters in carbon emissions studies than other models^29^. Therefore, we further performed a GWR model to examine the spatial heterogeneity in the influence of urban spatial structure elements. The model results are shown in Table 5. The adjusted R-squared was 0.7702, and the AICc was 96.8374, both better than those of the OLS regression; the standardized residuals from Global Moran’s I test showed a z-score of 1.081314, indicating that there is no spatial autocorrelation (Table 6). These results indicate that the impact of each spatial structure indicator on carbon emissions per unit of GDP is affected by geospatial location and that the GWR model can provide an in-depth analysis of the influence of each indicator based on OLS. The spatial distribution results of the GWR regression coefficients for each spatial structure element are provided in Fig. 7.

**Table 5:** GWR regression analysis result.

R^2^	0.7702
AdjR^2^	0.7035
AICc	96.8374
Sigma-squared	0.0828
Sigma-squared MLE	0.0642
Effective degrees of freedom	133.5336

**Table 6:** Global Moran’s I of GWR.

	GWR
Moran’s Index	0.074611
Expected Index	− 0.005848
Variance	0.005537
z-score	1.081314
p-value	0.279558

The results show that the influence of each spatial structure element on carbon emissions per unit of GDP varies significantly in different zones of the entire area.

The influence of most of the elements remains the same in terms of direction, but the intensity varies by region: the influence of fastway length gradually decreases from the northern mountainous region to the southern region; the influence of POI density decreases from the southeast to northwest; the influence of city road length gradually decreases from the north to the east; the influence of distance to the nearest transit station gradually decreases from the northwest to the southeast; and the influence of land use mix decreases from the edge of the city to the center. Construction site area is positively correlated with carbon emission efficiency in the southeast but is negatively correlated in the northwest. The station density is negatively correlated in the south, with its influence gradually decreasing from southwest to southeast, and gradually change into positively correlated in the north. The population density is negative and diminishing from the northern mountains to the southern regions, while positive in the southwest.

Some recent studies have found that factors such as population size in different parts of China and urban agglomerations can have a differential impact on carbon emissions by sector^28,30,31^. Sometimes positive and negative effects on emissions coexisted^28^. The findings of this paper further suggest that, similar to larger-scale studies, this differential impact on carbon emissions is equally present within cities.

**Figure 8:**
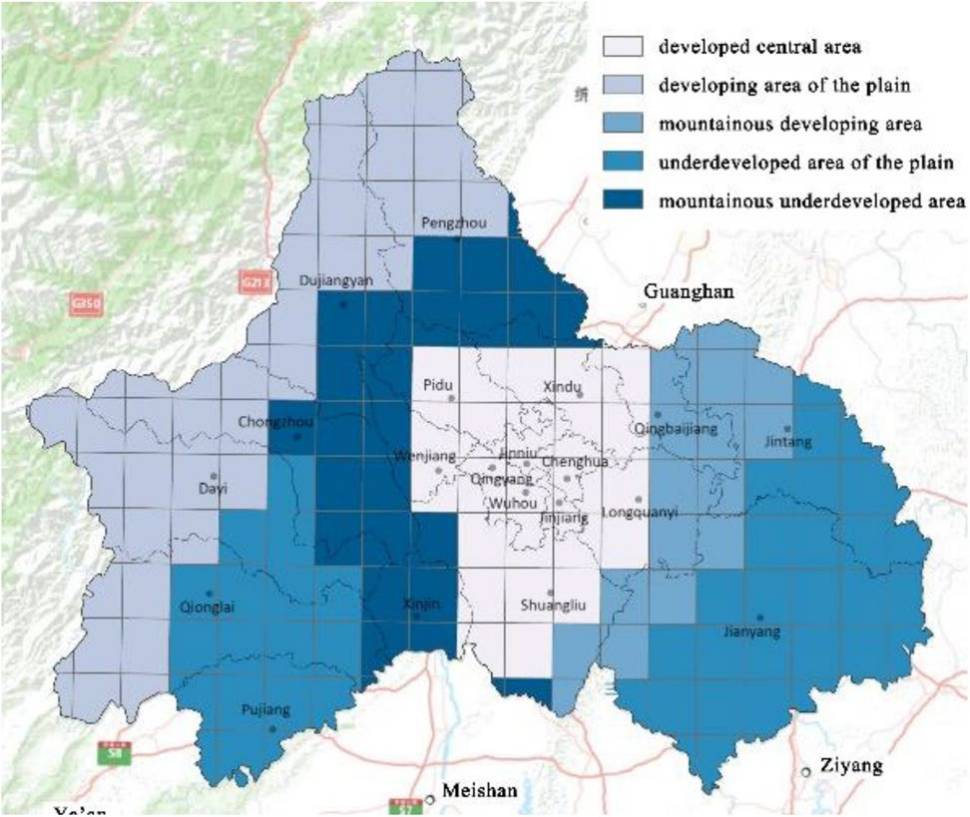
The city of Chengdu is divided into developed central area, developing area of the plain, mountainous developing area, underdeveloped area of the plain, and mountainous underdeveloped area by GDP and topography. (Note: ArcGIS Pro, Version 3.0.2, ESRI was used to create this figure).

**Figure 9:**
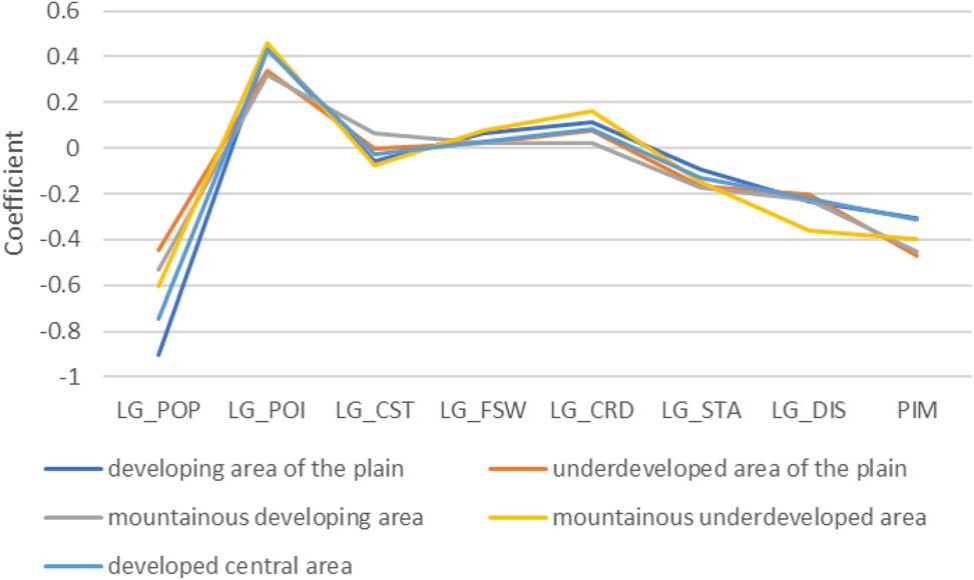
A chart to show the average influence coefficient of different urban spatial structure elements in each zone.

The differential impact is closely related to urbanization level, regional urban construction, economic development, and topography^32^. To explore more deeply the influence of urban spatial structure elements on carbon emission efficiency in different areas of the Chengdu megacity, we divided the city into five zones by GDP and topography in Chengdu: developed central area, developing area of the plain, mountainous developing area, underdeveloped area of the plain, and mountainous underdeveloped area (Fig. 8). We also calculated the average impact coefficient of different spatial structure elements in each area according to the GWR results, as shown in Fig. 9. It is important to note that higher carbon emissions per unit of GDP indicate lower carbon emission efficiency. Therefore, the effect of different spatial structure elements on carbon emission efficiency is the opposite of their influence on carbon emissions per unit of GDP. Based on the results, we analyzed the specific situation of each subdivision.

#### Developed central area

The developed central area is located in the main urban area of Chengdu, which has the fastest economic development rate in the city. In this area, higher population density, construction site area, land use mix, distance to the nearest transit station, and station density have significant positive effects on carbon emission efficiency. Among these, population density has the most apparent effect, and construction site area has the weakest positive influence. Correspondingly, POI density, city road length, and fastway length have a negative influence on carbon emission efficiency, with the most significant effect coming from the POI density.

Within the developed central area, the urban construction land area is saturated and the industry is mainly tertiary. With the out-migration of primary and secondary industries, the industrial types in the centrally developed areas rely less on goods transportation and more on commuter transportation. Therefore, the construction land and expressway length have little influence on carbon emission efficiency, while the density of bus stops and the distance to the nearest transportation station have an evident effect. For low-carbon construction, optimization of the urban public transportation system and decongestion of dense urban functions in the central developed area can focus factors for carbon reduction.

#### Developing area of the plain

This developing area of the plain is located in the western plain of Chengdu Central City. The increase in population density, construction site area, land use mix, distance to the nearest transit station, and density of station density resulted in a corresponding increase in carbon emission efficiency. Among them, the construction site area, distance to the nearest transit station, and station density had a more pronounced effect in the plain than in the central developed areas. Among the three elements (POI density, city road length, and fastway length) that had a negative influence on carbon emission efficiency, both road elements had a greater influence than those in the central developed areas.

The developing areas of the plain are in the stage of receiving the transfer of high-energy-consuming and low-output-value industries from urban centers, and the good transportation conditions owing to the developed road network have ushered in industrial agglomeration, causing high concentrations of carbon emissions. Such industrial transfer is an inevitable part of urban development; therefore, reasonably organizing the industrial structure, improving the utilization rate of waste energy, and reducing energy waste are the main factors to be addressed in the future for developing area of the plain.

#### Mountainous developing area

The mountainous developing area mainly includes the eastern Longquan Mountain area. The elements that positively affect carbon emission efficiency in this area include population density, land use mix, station density, and distance to the nearest transit stop. The POI density and construction land area had an evident negative effect on carbon emission efficiency. Unlike in other zones, city road length and expressway length had no significant effects.

This area is a long-developed automotive industry zone in Chengdu, with clustered industries, and a more developed upstream and downstream industrial system. At present, compared to the developing area of the plain, it receives a lower proportion of high-energy-consuming and low-output-value industries that are moving out of the urban center. As a result, the industrial agglomeration caused by the transportation system is smaller. The area’s developed industrial base resulted in a rapid growth of population. However, affected by the terrain, the population is highly concentrated in a relatively flat area near the main city, and the infrastructure is underdeveloped, resulting in low carbon emission efficiency. During its future development, the energy efficiency of industries, and optimization of the regional public transportation systems, and public service facilities should be the focus for higher carbon emission efficiency.

#### Underdeveloped area of the plain

The situation in the underdeveloped areas of the plain is similar to that in the developing area. Population density, station density, and distance to the nearest transit station are positively correlated with carbon emission efficiency, with population density having the weakest influence relative to other zones, and land use mix having the strongest influence relative to other zones. The POI density, fastway proportion, and city road proportion are negatively correlated, and their influence are relatively weak. In addition, the area of construction site area had no significant influence on this zone.

Underdeveloped areas of the plain are less developed than other zones, and the overall construction and development are slow-paced. However, likely to experience industrial transfer in future development, this area should be subject to transport network improvement, concentrated construction land utilization, and improvement of overall infrastructure. This underdeveloped area provides an opportunity for the application of urban planning strategies that can increase the population concentration and land use intensity that improve carbon emission efficiency.

#### Mountainous underdeveloped area

The construction site area, station density, distance to the nearest transit station, and land use mix are positively correlated with carbon emission efficiency in mountainous underdeveloped area, and the influence of population density, construction site area, and distance to the nearest transit station are stronger than in other zones. The population density, POI density, fastway length, and city road length are negatively correlated with carbon emission efficiency in this zone.

The data show that the carbon emission efficiency of mountainous underdeveloped areas is closely related to transportation and that the carbon emission efficiency of zones with better transportation conditions is lower. This is mainly due to the inconvenience of road construction in mountainous areas, thus high-energy-consuming secondary industries cannot be arranged in these areas as they require developed road networks. In other zones, carbon emissions mainly come from the primary and tertiary industries represented by rural tourism, with relatively high carbon emission efficiency. Increasing the degree of population density in mountainous areas, forming large-scale villager concentrations, improving infrastructure, and expanding public transportation routes and station settings between different zones can help carbon reduction in further development.

## Conclusion

The construction of low-carbon cities is an important goal in sustainable development. The efficiency of urban carbon emissions is influenced by multidimensional urban spatial structure elements^19^; this influence varies for different types of urban areas.

This study used the case of Chengdu to explore the variability of the influence of different urban spatial structure elements on carbon emission efficiency in different zones of the megacity. First, we measured and analyzed spatial carbon emission efficiency and multidimensional urban spatial structure elements by constructing a high-resolution grid. Then, we used OLS and GWR regression analyses to identify the main elements affecting carbon emission efficiency. Next, we discuss the spatial heterogeneity in the effect of each element by zone. The findings of this study are as follows.

1. The overall carbon emission efficiency of Chengdu city is high in the center and low in the surrounding areas. Two of the highest values appear in the vicinity of the Longquan Mountain Range of the main urban area of Chengdu and the mountainous area of western Chengdu.
2. The carbon emission efficiency in Chengdu is closely related to urban density, functional mix, land use type, and traffic structure. Under the combined effect of multiple elements, POI density, fastway length, and city road length were negatively correlated with carbon emission efficiency, while population density, construction site area, station density, distance to the nearest transit station, and land use mix were positively correlated with carbon emission efficiency.
3. The impact of each spatial structure element shows differences in different zones of Chengdu, thus differentiated development strategies are necessary for each zone to increase emissions efficiency. The developed central area should further optimize the urban public transportation system and decentralize the overly dense urban functions; the developing area of the plain should rationally organize the industrial structure, improve the utilization rate of waste energy, and reduce energy waste. The mountainous developing area should promote the energy efficiency of industries and further optimize the regional public transportation system and public service facilities. The underdeveloped areas of the plain should select locations to promote population concentration and intensive land use to improve the efficiency of existing carbon emissions and provide more adequate conditions for future development; the mountainous underdeveloped areas should form a relatively large concentration of villagers, carry out overall infrastructure construction, and expand public transport construction between the points.

The findings show that for different zones within cities, urban spatial structure elements have different impacts on carbon emission efficiency, for which a higher resolution grid study can effectively express and analyze. Due to the limitation of top-down data acquisition, this study has not yet conducted a more detailed division within the city, for example industrial agglomeration and residential agglomeration. For further studies, a higher-resolution grid can be considered for a more detailed discussion of different functional areas of the city through bottom-up data acquisition, which would provide more practical guidance for the specific design of the city.

## Data Availability

The datasets used and/or analysed during the current study available from the corresponding author on reasonable request.
